# A nanoluciferase biosensor to investigate endogenous chemokine secretion and receptor binding

**DOI:** 10.1016/j.isci.2020.102011

**Published:** 2020-12-30

**Authors:** Carl W. White, Laura E. Kilpatrick, Kevin D.G. Pfleger, Stephen J. Hill

**Affiliations:** 1 Cell Signalling and Pharmacology Research Group, Division of Physiology, Pharmacology & Neuroscience, School of Life Sciences, University of Nottingham, Nottingham NG7 2UH, UK; 2 Centre of Membrane Proteins and Receptors, University of Birmingham and University of Nottingham, The Midlands, UK; 3 Harry Perkins Institute of Medical Research and Centre for Medical Research, The University of Western Australia, QEII Medical Centre, Nedlands, WA 6009, Australia; 4 Australian Research Council Centre for Personalised Therapeutics Technologies, Australia; 5 School of Pharmacy, Biodiscovery Institute, University of Nottingham, Nottingham, NG7 2RD, UK; 6 Dimerix Limited, Nedlands, WA 6009, Australia

**Keywords:** Techniques in Genetics, Molecular Interaction, Biotechnology

## Abstract

Secreted chemokines are critical mediators of cellular communication that elicit intracellular signaling by binding membrane-bound receptors. Here we demonstrate the development and use of a sensitive real-time approach to quantify secretion and receptor binding of native chemokines in live cells to better understand their molecular interactions and function. CRISPR/Cas9 genome editing was used to tag the chemokine CXCL12 with the nanoluciferase fragment HiBiT. CXCL12 secretion was subsequently monitored and quantified by luminescence output. Binding of tagged CXCL12 to either chemokine receptors or membrane glycosaminoglycans could be monitored due to the steric constraints of nanoluciferase complementation. Furthermore, binding of native CXCL12-HiBiT to AlexaFluor488-tagged CXCR4 chemokine receptors could also be distinguished from glycosaminoglycan binding and pharmacologically analyzed using BRET. These live cell approaches combine the sensitivity of nanoluciferase with CRISPR/Cas9 genome editing to detect, quantify, and monitor binding of low levels of native secreted proteins in real time.

## Highlights

• Quantification of endogenous CXCL12 secretion using CRISPR/Cas9 genome-editing
• A NanoBRET approach to investigate CXCL12-receptor binding
• Detection of CXCL12-GAG interactions using nanoluciferase complementation

## Graphical abstract

**Figure:**
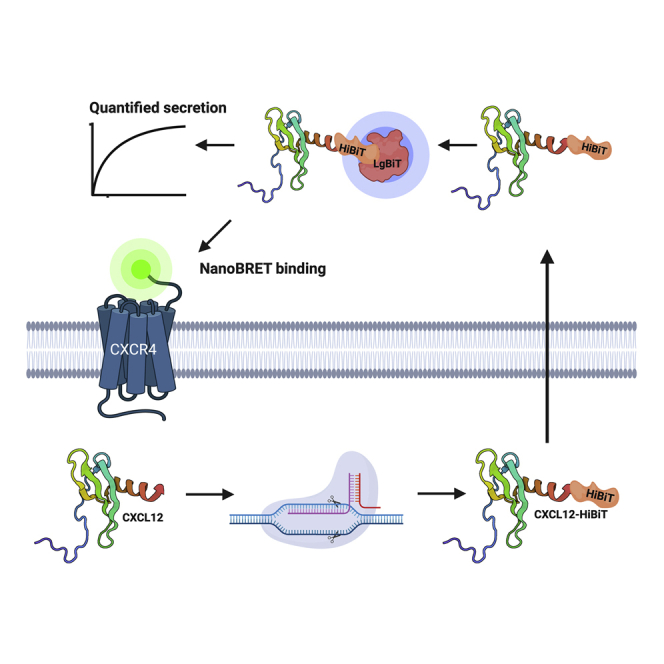

## Introduction

Secreted peptides and proteins are critical for cellular communication, with changes in expression, secretion, and subsequent binding to their cellular targets mediating numerous (patho)-physiological cellular responses. Chemokines are a family of small cytokines secreted by cells that bind and activate G protein-coupled receptors, resulting in immune cell migration; cell differentiation and development; and cancer metastasis (Hughes and Nibbs, 2018). The function of chemokines is controlled at the transcriptional as well as the post-translational level. For example, numerous factors are known *in vivo* to induce the expression and production of chemokines to recruit immune cells to inflamed tissue, whereas secreted chemokines bind membrane-bound proteoglycans to form chemotactic gradients that guide immune cell migration (Proudfoot et al., 2017; Stone et al., 2017). Investigating and quantifying chemokine ligand secretion from cells, as well as interactions with glycosaminoglycans (GAGs) found on proteoglycans and their receptors, is therefore important to properly understand chemokine regulation and function.

CXCL12, also known as stromal derived factor, is a prototypical chemokine that binds CXCR4 to mediate immune cell migration and cellular differentiation (Busillo and Benovic, 2007) and is a known biomarker for a number of cancers (Samarendra et al., 2017). Like many secreted proteins, methods to quantify chemokine expression rely on monitoring mRNA transcript levels or mass spectrometry and immunoassay assays such as ELISA and western blotting to determine protein levels. However, these methods have limited sensitivity for detecting poorly expressed proteins and, in the case of immunoassays, rely on the availability of sensitive and selective antibodies (McDonough et al., 2015). Such approaches are also relatively low throughput and lack the temporal fidelity required to investigate chemokine secretion in a real-time manner. In addition, these methods only inform on the expression of the chemokine without imparting knowledge of downstream signaling. Detailed pharmacological analysis of chemokine binding to their cellular targets, i.e. chemokine receptors, proteoglycans or GAGs, is therefore commonly monitored separately using approaches such as radio- or fluorescent ligand binding (Stoddart et al., 2016) and surface plasmon resonance assays (Du et al., 2016). Such assays performed in live cell formats may struggle to differentiate between the different binding sites, whereas those configured to look at specific interactions, e.g. surface plasmon resonance, are performed with purified receptor and therefore occur in more artificial environments (Du et al., 2016). Furthermore, exogenous chemokine is used rather than chemokines secreted from cells and expressed under endogenous promotion.

Fusion of a luciferase to a protein of interest has allowed a wide range of biological effects to be investigated by bioluminescent technologies such as luciferase complementation and bioluminescence resonance energy transfer (BRET) (Kaskova et al., 2016). Luciferase reporters have high signal-to-noise ratios, therefore providing highly sensitive detection of low abundance proteins as well as excellent quantitation over an extensive linear concentration range (Dixon et al., 2016; Hall et al., 2012). Previously, bioluminescence approaches using full-length or split Gaussia luciferase have been used to investigate CXCL12 binding to CXCR4 and to the atypical chemokine receptor ACKR3 both *in vitro* and *in vivo* (Luker et al., 2009, 2012; Luker and Luker, 2014). However, these studies used either purified luciferase-tagged CXCL12 or cells secreting exogenous luciferase-tagged CXCL12 to monitor binding by luciferase complementation or changes in luminescence, rather than endogenously expressed CXCL12. More recently, we have demonstrated that ligand binding to CXCR4 and ACKR3 tagged with the nanoluciferase (NLuc) can be monitored in live cells using CXCL12 labeled with AF647 and NanoBRET (White et al., 2020). It has been shown that a split version of NLuc can be used to investigate peptide ligand binding to relaxin peptide family receptor 3 and 4 by NLuc complementation (Wang et al., 2019). However, in these studies the peptide used to monitor these interactions was exogenously derived.

Taking advantage of the brightness of NLuc (Hall et al., 2012), we and others have used CRISPR/Cas9-mediated genome engineering to tag and then study genes and proteins expressed under endogenous promotion using either full-length or split NLuc. This has allowed changes in gene expression or protein levels to be measured and quantified (Lackner et al., 2015; Oh-Hashi et al., 2016; Schwinn et al., 2018), as well as ligand binding and internalization (Boursier et al., 2020; White et al., 2020), protein-protein interactions (White et al, 2017, 2020), post-translational modifications (Schwinn et al., 2018), and protein degradation (Riching et al., 2018) to be monitored in real-time live cell or lysed cell assays by NanoBRET or changes in luminescence for NLuc complementation. Although these studies using CRISPR/Cas9 genome editing and luciferase tags have principally investigated membrane bound and intracellular proteins, vascular endothelial growth factor (VEGFA), a secreted growth factor, was previously tagged with the small NLuc fragment (HiBiT) (Schwinn et al., 2018) and expression measured in a lytic cell assay at a single time point, indicating the potential for use of this approach to monitor secreted chemokines. Biosensors capable of investigating both ligand secretion and ligand binding of endogenously expressed proteins will be highly useful to understand cellular signaling. Using CRISPR/Cas9-mediated genome engineering, here we report a live cell assay that can be used to monitor and quantify secretion of proteins expressed under endogenous promotion by luciferase complementation as well as chemokine ligand binding by BRET in real-time and in live cells.

## Results and discussion

### Genome editing of CXCL12

CXCL12 is an essential chemokine secreted endogenously by many cells including the immortalized HEK293 cell line that is commonly used to investigate receptor pharmacology. However, real-time live cell assays to quantify endogenous CXCL12 levels are lacking. Here, we sought to develop such an assay to monitor expression and secretion of endogenous CXCL12 from HEK293 cells and to do so we first used CRISPR/Cas9 genome editing to append the small 11-amino acid fragments of NLuc, HiBiT, to the C-terminus of CXCL12. The use of a small NLuc fragment rather than full-length NLuc simplifies and improves the efficiency of the genome-editing process in cells. Indeed, we found homozygous HiBiT insertion into the native *CXCL12* locus (Figure S1), a low probability event in non-diploid HEK293 cells. The minimal size also reduces the potential of the tag to perturb the function of CXCL12, with the placement of the HiBiT tag on the C-terminus of CXCL12 based on the knowledge that the N-terminus is critical for CXCR4 activation (Crump et al., 1997) and previous fusion of Gaussia luciferase to CXCL12 in overexpression models did not impede function (Luker et al., 2009). Indeed, as noted below the pharmacology of CXCL12-HiBiT was similar to that seen previously for CXCL12-AF647 (White et al., 2020). This strategy using HiBiT rather than full-length NLuc also limits the observable signal to secreted (extracellular) CXCL12 due to the cell impermeant nature of the 18 kDa NLuc fragment (LgBiT) used for luciferase complementation.

### Quantification of ligand secretion

**Figure 1:**
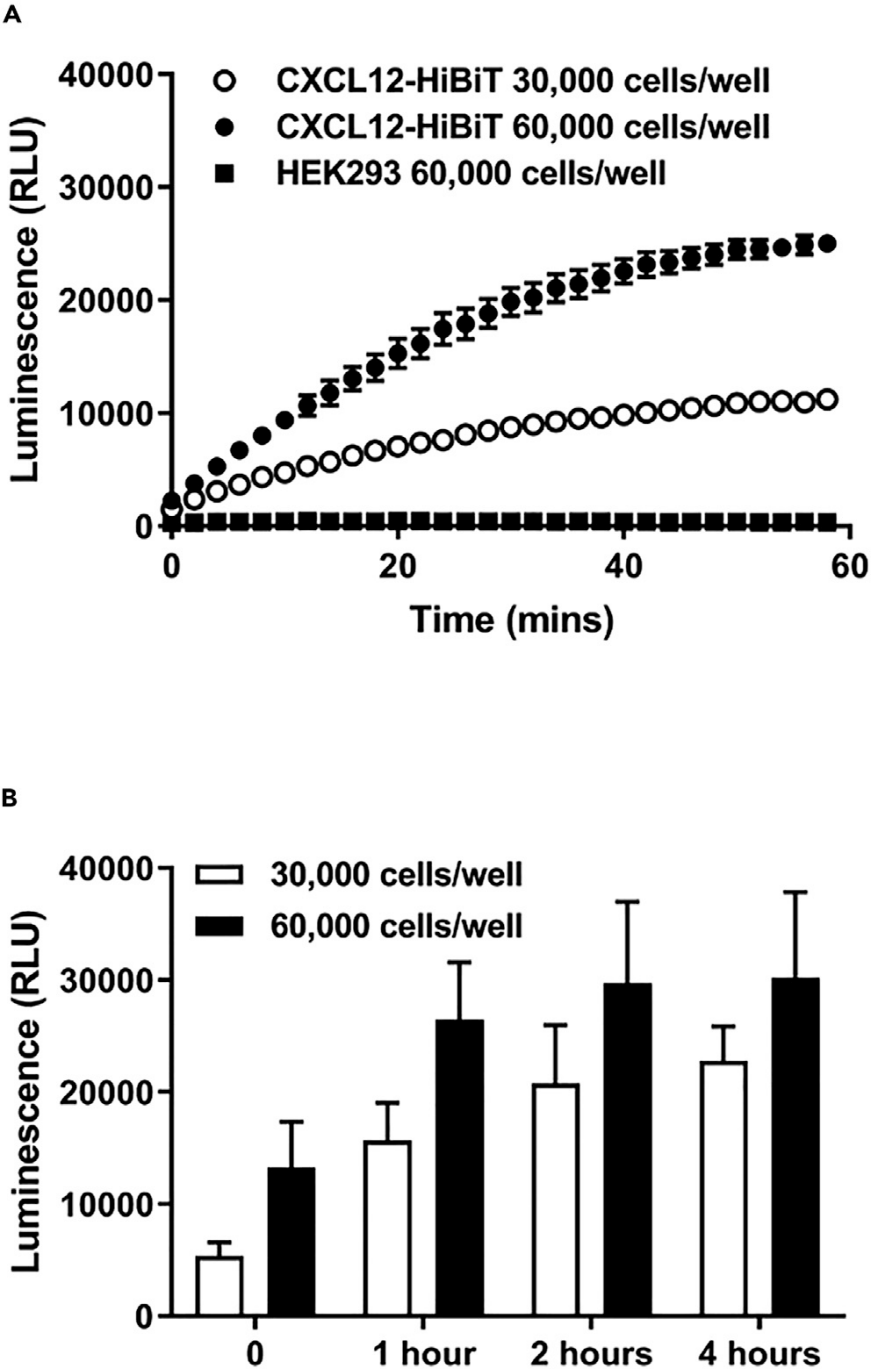
Investigation of CXCL12-HiBiT secretion from genome-edited HEK293 cells (A) Kinetic analysis of luminescence generated post-wash from HEK293 cells expressing genome-edited CXCL12-HiBiT plated at 30,000 (white circles) or 60,000 (black circles) cells/well or wild-type HEK293 cells (squares) immediately following LgBiT (30 nM) addition. (B) Effect of cell number and post-wash incubation time on luminescence generated by addition of LgBiT (30 nM) to HEK293 cells expressing genome-edited CXCL12-HiBiT. Points and bars are (B) mean ± s.e.m. of three experiments performed in triplicate or are (A) representative of three experiments.

In live HEK293 cells expressing genome-edited CXCL12-HiBiT incubated with LgBiT (30 nM), we observed a gradual increase in luminescence (Figure 1A) over the course of 1 h, and increasing either cell number or incubation time further augmented the increase in luminescence (Figures 1A and 1B), indicating continuous CXCL12-HiBiT secretion. At longer time points, 2 and 4 h, we observed that the increase in luminescence plateaued (Figure 1B). This is likely to be due to CXCL12-HiBiT secretion reaching equilibrium with CXCL12-HiBiT sequestration and/or internalization following binding to glycosaminoglycans and endogenous CXCR4 expressed in HEK293 cells, respectively. Indeed, CXCR4 can internalize CXCL12 (Hoffmann et al., 2012) and as noted below binding of CXCL12-HiBiT to glycosaminoglycans reduces the luminescent output due to steric constraints. However, our initial data (Figure 1) report relative changes in expression rather than provide absolute quantification of secreted protein. Such measures can be made using traditional immunoassays and are important for pharmacological evaluation of ligand function. To address this, we sought to quantify expression through correlation with luminescent output.

**Figure 2:**
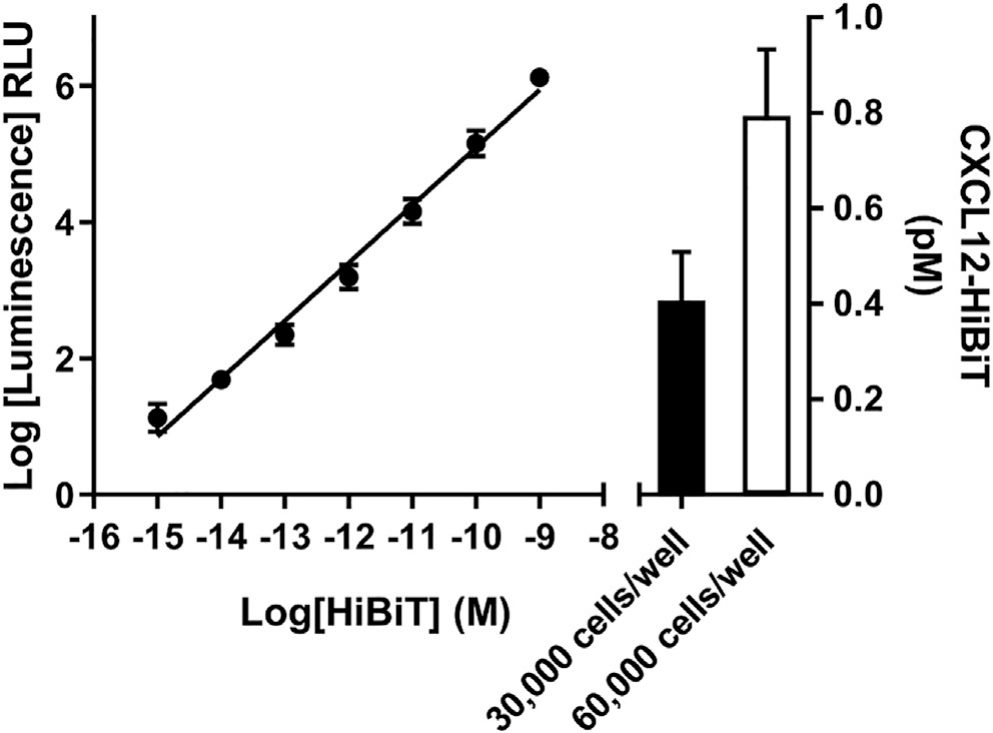
Quantification of CXCL12-HiBiT expression in genome-edited HEK293 cells Luminescence generated from purified LgBiT (100 nM) incubated with increasing concentrations of purified HiBiT-Halotag (1 fM - 1 nM) was used to construct a linear standard curve to quantify CXCL12-HiBiT expression by linear regression in wells seeded with either 30,000 (black bar) or 60,000 (white bar) genome-edited HEK293 cells. Points and bars are mean ± s.e.m. of six experiments performed in triplicate.

Previous work has shown that complementation of the two NLuc fragments (HiBiT with LgBiT) is linear, with luminescence extending over eight orders of magnitude (Schwinn et al., 2018). Here we took advantage of this relationship to quantify secreted CXCL12-HiBiT expression from our genome-edited live HEK293 cells (Figure 2). At 2 h post-wash, when CXCL12 secretion had largely plateaued, we observed CXCL12-HiBiT expression to be 0.41 ± 0.10 pM (n = 6) and 0.79 ± 0.14 pM (n = 6) in a 96 well plate seeded with 30,000 and 60,000 cells/well, respectively (Figure 2). Although the current data demonstrate absolute quantification, this is of luciferase-tagged CXCL12 under endogenous promotion rather than endogenous CXCL12, and CRISPR/Cas9 tagging of native proteins may alter expression (Khan et al., 2019; White et al., 2020). Although no such effects were seen previously with insertion of small NLuc tags (White et al., 2020), changes in expression relative to wild-type cells expressing untagged CXCL12 due to tagging or genetic rewiring following prolonged passage may need to be considered. Commercial immunoassays for CXCL12 detection regularly have a sensitivity of >1 pg/mL (∼0.1 nM) and normal working ranges of 10–1,000 pg/mL. In contrast here, the sensitivity of the luminescent approach allowed detection of natively expressed CXCL12-HiBiT that is at the absolute lower end of the sensitivity of immunoassays and was approximately 1000-fold more sensitive than these approaches with measurable signal in the low fM range. Moreover, we observed luminescence output that was linear over seven orders of magnitude, which exceeds the typical dynamic range (2–3 orders of magnitude) seen with standard immunoassays. These results demonstrate the applicability of coupling genome editing with nanoluciferase tags to monitor secreted protein levels in real time and in live cells. Importantly, these approaches overcome the low-throughput non-live cell limitations of common techniques used to quantify protein expression, such as ELISA and western blotting.

### NanoBRET ligand binding

**Figure 3:**
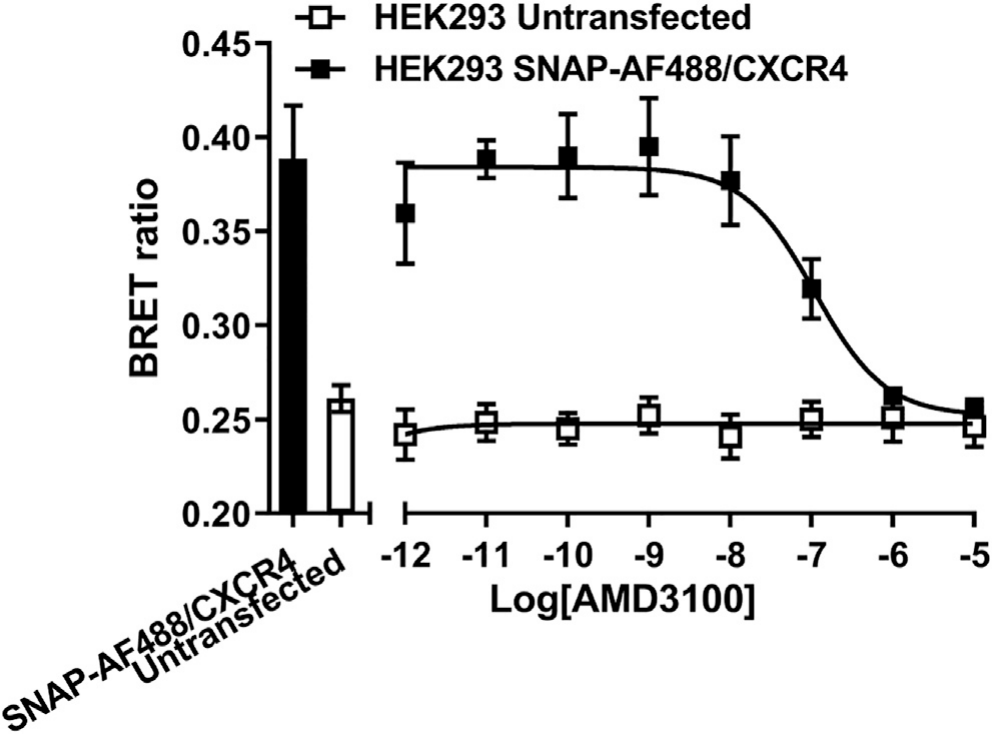
Displacement of genome-edited CXCL12-HiBiT binding to SNAP/CXCR4 observed by NanoBRET HEK293 cells expressing genome-edited CXCL12-HiBiT, co-cultured with wild-type HEK293 cells (white squares) or HEK293 cells transiently transfected with SNAP/CXCR4 (black squares), were incubated in the absence or presence of increasing concentrations of AMD3100 (1 pM–10 μM). CXCL12-HiBiT was complemented with purified LgBiT (30 nM) and SNAP/CXCR4 labeled with cell impermeant Alexa Fluor 488. Bars represent basal BRET in the absence of AMD3100. Bars and points represent mean ± s.e.m. of five individual experiments performed in duplicate.

Exogenous CXCL12 tagged with full-length Gaussia luciferase have been used to investigate ligand binding at CXCR4 and ACKR3, with fusion of the luciferase having limited effects on CXCL12 function (Luker et al., 2009). To ensure that CXCL12 tagged with HiBiT retained functionality and was not degraded following secretion, we established a NanoBRET ligand binding configuration that should be widely applicable to chemokines and other secreted proteins such as growth factors. Here, HEK293 cells expressing genome-edited CXCL12-HiBiT were co-cultured with wild-type HEK293 cells or HEK293 cells transiently transfected with SNAP/CXCR4. Binding of CXCL12-HiBiT to CXCR4 brings the donor luciferase (once it has been complimented with cell impermeant purified LgBiT) on the ligand and a fluorescent reporter on the receptor into close proximity, thereby increasing the BRET ratio that can be measured and inferred as ligand binding. Compared with coincubation with wild-type HEK293 cells, we observed an increase in BRET between CXCL12-HiBiT (complemented with purified LgBiT) and SNAP/CXCR4 labeled with cell impermeant Alexa Fluor 488 that was displaced in a concentration-dependent manner by the CXCR4 antagonist AMD3100 at the anticipated affinity (Figure 3, pIC_50_ = 6.97 ± 0.10, n = 5). Notably, the pharmacology of CXCL12-HiBiT binding to SNAP/CXCR4 is similar to that observed previously using fluorescently tagged CXCL12-AF647 (White et al., 2020), further demonstrating the functionality of CXCL12-HiBiT secreted from these cells. Taken together, these data show that the genome-edited CXCL12-HiBiT is both secreted and capable of binding to CXCR4 exogenously expressed in neighboring cells.

In addition to investigating binding using CXCL12 tagged with full-length luciferase, purified or secreted CXCL12 tagged with split-Gaussia luciferase has been used to probe CXCR4 or ACKR3 tagged with the corresponding Gaussia luciferase fragment *in vitro* and *in vivo* (Luker and Luker, 2014). More recent studies have used split-NLuc complementation to investigate ligand binding at the relaxin-3 receptor (Wang et al., 2019) or binding of a nanobody (VUN400-HiBiT) to CXCR4 tagged with split-NLuc fragments (Soave et al., 2020). However, here we used NanoBRET rather than luciferase complementation to investigate ligand binding. NanoBRET confers the advantage of reducing any confounding effects due to the affinity of the luciferase complementation, particularly if investigating low-affinity ligand-receptor interactions. Moreover, the high distance dependence of energy transfer in NanoBRET assays distinguishes binding of CXCL12 at receptors from non-receptor binding such as at glycosaminoglycans (GAGs). Indeed, as discussed below, this may be a useful consideration because interactions with GAGs both regulate and add complexity to chemokine signaling (Proudfoot et al., 2017). Finally, traditional NanoBRET binding assays where the receptor is tagged with NLuc requires suitable fluorescent ligands that are not always readily available. Our approach achieves the benefits of NanoBRET ligand binding without the need to generate complex fluorescent proteins/probes that may require recombinant production.

### Detection of interactions between chemokine and GAG

We have previously reported that knockout of CXCL12 from HEK293 cells decreases constitutive CXCR4 internalization, suggesting that sufficient endogenous CXCL12 is secreted to activate and internalize CXCR4 (White et al., 2020). However, the apparent concentration of CXCL12-HiBiT that we observed is approximately 300- to 1000-fold lower than the reported EC_50_ for CXCL12-mediated CXCR4 G protein signaling (Levoye et al., 2009). It is known that ligand concentration within a plate-based cellular assay is unlikely uniform (Gherbi et al., 2018) and that secreted chemokines can be concentrated/localized at the cell surface by binding to GAGs, which increases the effective ligand concentration near the receptor (Proudfoot et al., 2017). We have shown previously that when NLuc fragments are fused to proteins, steric constraints can modulate the affinity of complementation (White et al., 2020), whereas binding of chemokines to GAGs provides protection from proteolytic degradation as well as facilitates chemokine oligomerization and clustering (Proudfoot et al., 2017). We therefore hypothesized that CXCL12 binding to GAGs would impart such constraints on NLuc complementation and provide a mechanism to determine if GAG-mediated accumulation was indeed occurring.

**Figure 4:**
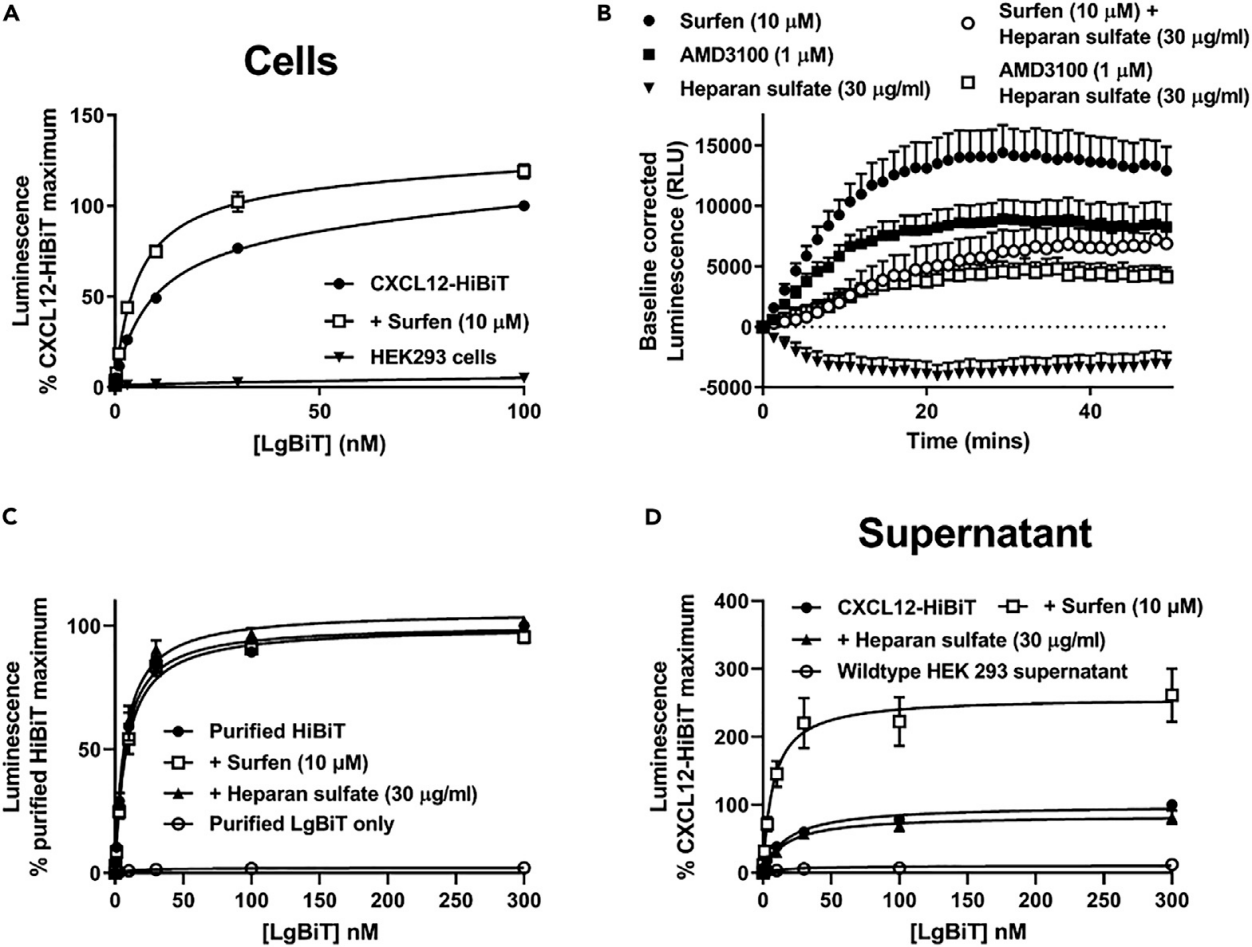
Monitoring CXCL12-glycosaminoglycan interactions by changes in luminescent output (A) HEK293 cells expressing genome-edited CXCL12-HiBiT or wild-type HEK293 cells (downward triangles) were incubated with increasing concentrations of purified LgBiT in the absence (black circles) or presence of surfen (10 μM, white squares). (B) Kinetic analysis of the effect of AMD3100 (1 μM, black square), surfen (10 μM, black circle), heparan sulfate (30 μg/mL, downward triangle), AMD3100 (1 μM) plus heparan sulfate (30 μg/mL, white square), or surfen (10 μM) plus heparan sulfate (30 μg/mL, white circle) on the baseline-corrected luminescence generated by HEK293 cells expressing genome-edited CXCL12-HiBiT in the presence of 30 nM LgBiT. (C) Purified HiBiT (1 nM) was incubated with increasing concentrations of purified LgBiT in the absence (black circles) or presence of surfen (10 μM, white squares) or heparan sulfate (30 μg/mL, black triangles). (D) Supernatants from HEK293 cells expressing genome-edited CXCL12-HiBiT or wild-type HEK293 cells (white circles) were incubated with increasing concentrations of purified LgBiT in the absence (black circles) or presence of surfen (10 μM, white squares) or heparan sulfate (30 μg/mL, black triangles). (A, C, and D) points represent percentage of the maximum luminescence ±s.e.m. generated by complementation of genome-edited CXCL12-HiBiT LgBiT in the vehicle control from four to six individual experiments performed in duplicate. (B) Points represent mean ± s.e.m. of five individual experiments performed in duplicate. See also Figure S2.

To test this, we first determined the affinity of NLuc complementation in our HEK293 cells expressing genome-edited CXCL12-HiBiT. We found that in live cells the affinity of NLuc complementation (CXCL12-HiBiT with LgBiT) was reduced (K_d_ = 11.6 ± 1.48 nM, mean ± s.e.m., Figure 4A) compared with the affinity (∼700 pM) for purified NLuc fragments reported in the literature (Dixon et al., 2016) and the affinity we observed using our methodologies (White et al., 2020), indicating that fusion of HiBiT to CXCL12 modulated complementation. Next we used a small molecule glycosaminoglycan inhibitor (Schuksz et al., 2008) surfen (10 μM) to pharmacologically disrupt CXCL12-GAG binding and observed a small increase in the affinity of CXCL12-HiBiT-LgBiT complementation compared with the vehicle control (Figure 4A; K_d_ = 5.93 ± 0.53 nM, mean ± s.e.m., n = 6, p < 0.05), as well as an increase in the luminescence output (Figures 4A and 4B). In contrast, incubation with exogenous heparan sulfate (30 μg/mL), a major glycosaminoglycan, decreased luminescence and attenuated the surfen-mediated responses (Figure 4B, p < 0.01, n = 5). In addition, we also observed that incubation of cells with AMD3100 (1 μM, Figure 4B) increased luminescence output, indicating that CXCL12-HiBiT binds to both endogenous GAGs and CXCR4 expressed in HEK293 cells. Supporting the ligand-induced change in complementation affinity being due to a specific interaction between CXCL12-HiBiT and GAGs, neither surfen (10 μM; K_d_ = 8.24 ± 0.87, mean ± s.e.m, n = 4) nor heparan sulfate (30 μg/mL; K_d_ = 7.16 ± 0.66, mean ± s.e.m, n = 4) modulated the affinity of purified HiBiT and purified LgBiT complementation compared with vehicle control (Figure 4C; K_d_ = 7.15 ± 0.66 nM, mean ± s.e.m, n = 4), whereas we have previously reported that AMD3100 also has no effect on this interaction (White et al., 2020). Therefore, the differences in complementation affinity between HiBiT and LgBiT are, in part, due to conformational differences of CXCL12-HiBiT when bound to GAGs and/or in oligomeric forms compared with when found in a free state.

Finally, in a subset of experiments we also investigated whether CXCL12-HiBiT was also bound to secreted GAGs. In these studies, we observed that the affinity of NLuc complementation (CXCL12-HiBiT with LgBiT) was also reduced compared with the affinity of purified NLuc fragments in supernatants collected from genome-edited HEK293 cells (Figure 4D; K_d_ = 17.47 ± 2.13 nM, mean ± s.e.m., n = 6). We also observed that surfen (10 μM; K_d_ = 7.46 ± 2.27 nM, mean ± s.e.m., n = 4) increased the affinity of complementation as well as luminescence output compared with the vehicle control but that AMD3100 (1 μM; Figure S2, n = 4) had no effect. This demonstrates that CXCL12-HiBiT secreted from HEK293 cells can bind to secreted/soluble GAGs and confirmed that no CXCR4, and therefore cells, were present in the supernatants. Notably, heparan sulfate (30 μg/mL; K_d_ = 16.15 ± 3.83 nM, mean ± s.e.m., n = 4) did not modulate CXCL12-HiBiT–LgBiT complementation affinity or luminescent output in CXCL12-HiBiT containing supernatants compared with vehicle control. This suggests that most secreted CXCL12-HiBiT in the supernatants is bound to GAGs, which prevents further interactions between CXCL12-HiBiT and exogenously applied heparan sulfate. Comparison of the affinity of CXCL12-HiBiT-LgBiT complementation in live cells and supernatants (K_d_ = 11.6 ± 1.48 nM versus K_d_ = 17.47 ± 2.13 nM) further supports this, as in live cells both GAG bound and free CXCL12-HiBiT are likely present. Indeed, the decrease in luminescent output (Figure 4B) in the live cell assay caused by application of exogenous heparan sulfate is likely due to binding of newly secreted or free CXCL12-HiBiT rather than binding to existing extracellular GAG bound CXCL12-HiBiT.

Here we demonstrate that interactions between CXCL12 and GAG are occurring in cultures of HEK293 cells and that by monitoring changes in NLuc complementation these interactions can be investigated in a live cell assay. Although, as hypothesized, the results suggest a possible mechanism by which a cell may achieve sufficient accumulation of endogenous CXCL12 at or near the membrane to activate CXCR4, from the current study we are unable to differentiate CXCL12 binding to secreted versus membrane-bound GAGs. Nevertheless, GAG binding also protects chemokines from proteolytic degradation and therefore CXCL12-HiBiT binding to either secreted or membrane-bound GAGs would also facilitate extracellular ligand accumulation. Interestingly, our observations also suggest that once secreted into the media and free from the extracellular matrix, CXCL12-HiBiT is predominantly bound to GAGs. Within a cellular signaling context, interactions between secreted chemokines and GAGs may limit degradation as well as help maintain chemotactic gradients. Indeed, it is known that interactions between chemokine and GAG are important for chemotaxis *in vivo* (Crijns et al., 2020) and that soluble chemokines that activate leukocytes in the circulation prior to adhesion to the endothelium impair leukocyte adhesion and emigration (Ley et al., 1993). Finally, it is noteworthy that although HEK293 cells are a common model cell line used to study receptor function, the concurrent expression, membrane accumulation, and receptor activation of endogenously secreted ligands from these cells are underappreciated and may confound the interpretation of results.

### Summary and future directions

In summary, the genome-editing approach described here allows the secretion and binding of endogenous chemokines to GAGs or receptors to be readily inferred in live cell assays through changes in luminescence and/or NanoBRET ligand-binding assays. These approaches monitor continuous peptide secretion in real time and do not require the development of a selective and/or specific antibody. Importantly, binding specificity can be imparted using the NanoBRET modality that allows binding of secreted ligand to receptors to be monitored while effectively eliminating observable “off-target” binding to GAGs. Similar to many peptides, chemokine binding to GAGs *in vivo* is vital for modulating function; however, thus far these interactions have primarily been explored in artificial/purified non-cell systems or in cellular assays that poorly distinguish between multiple binding targets (Hamel et al., 2009), i.e. receptors and GAGs. It is therefore envisioned that these *in vitro* approaches will be broadly applicable to investigate secretion and binding of peptides and contribute to our understanding of chemokine-GAG binding and chemokine-mediated signaling in cells.

### Limitations of the study

All experiments in this study were performed in model HEK293 cells; therefore, the broad applicability of the approach to other secreted peptides will depend on the ability to use CRISPR/Cas9 to edit cells of interest. In general, fusion of HiBiT to a protein may alter protein stability or expression, and therefore, the amount of HiBiT-tagged CXCL12 quantified may differ from the true levels of endogenous CXCL12 in HEK293 cells. Finally, it was not possible by luciferase complementation to determine to which specific types of proteoglycans/GAGs that CXCL12-HiBiT binds, but rather it was inferred that such interactions are occurring. Such specific interactions may be able to be explored in the future using the NanoBRET-binding approach described here.

### Resource availability

#### Lead contact

Further information and requests for resources and reagents should be directed to and will be fulfilled by the Lead Contact, Stephen J Hill (stephen.hill@nottingham.ac.uk).

#### Materials availability

Materials developed from this study are available from the Lead author on reasonable request.

#### Data and code availability

This study did not generate datasets or code.

## Methods

All methods can be found in the accompanying Transparent Methods supplemental file.

## Supplemental information

Document S1.Transparent methods and Figures S1 and S2
