# Supplementary material for: A nanoluciferase biosensor to investigate endogenous chemokine secretion and receptor binding
Source: iScience. 2020 Dec 30;24(1):102011. doi: 10.1016/j.isci.2020.102011 (PMC7809502; doi:10.1016/j.isci.2020.102011)
Supplement: Document S1.Transparent methods and Figures S1 and S2 [file mmc1.pdf]

iScience, Volume *24*

Supplemental Information

A nanoluciferase biosensor to investigate endogenous chemokine secretion and receptor binding

Carl W. White, Laura E. Kilpatrick, Kevin D.G. Pfleger, and Stephen J. Hill

Supplemental Data

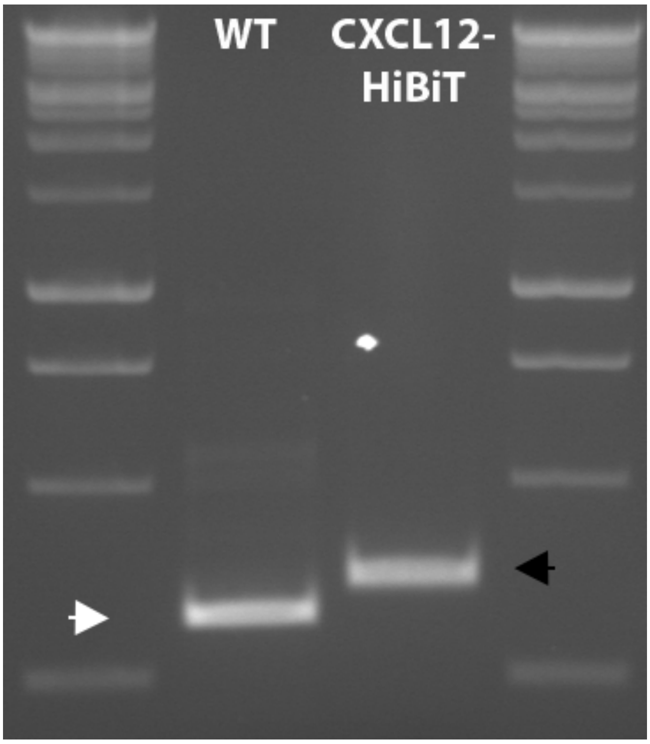

Figure S1: Genotyping of wildtype and genome-edited HEK293 cell lines, Related to Figure 1. PCR amplification using primers targeting the CXCL12 locus of genomic DNA extracted from wildtype (WT) HEK293 cells (**lane 2)** or HEK293 cells genome-edited to express CXCL12-HiBiT (**lane 3)**. Wildtype PCR product at 314bp indicated by white arrow and black arrow indicates PCR product of inserted tag at 359bp. Lanes 1 and 4: Promega 1Kb benchtop DNA ladder.

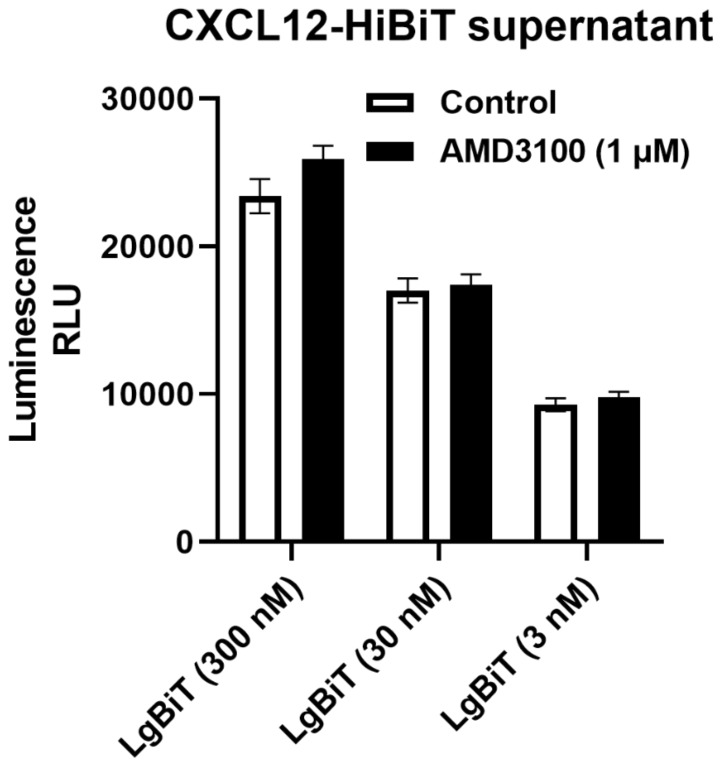

Figure S2: Effect of CXCR4 inhibition on luminescent output from supernatants collected from HEK293 cells expressing genome-edited CXCL12-HiBiT, Related to Figure 4. Supernatants were incubated with purified LgBiT (3 nM, 30 nM or 300 nM) in the absence (white bars) or presence of AMD3100 (1 μM, black bars). Bars represent luminescence in relative light units (RLU) ± s.e.m. generated by complementation of genome-edited CXCL12-HiBiT and LgBiT from four individual experiments performed in duplicate or triplicate.

Transparent Methods

Materials

AMD3100 was purchased from Selleckchem (USA). Heparan sulfate sodium salt from bovine kidney (heparan sulfate) and surfen hydrate (surfen) were from Sigma-Aldrich (United Kingdom). Furimazine, purified HiBiT (HiBiT-Halotag, control peptide) and purified LgBiT NLuc fragments were purchased from Promega (USA). Membrane impermeant SNAP-surface AF488 was purchased from New England Biolabs (United Kingdom). AMD3100 (10 mM) and heparan sulfate were dissolved in water. Surfen hydrate (10 mM) was dissolved in Dimethyl sulfoxide (DMSO). All further dilutions were performed in assay buffer containing 0.1% bovine serum albumin (BSA, Sigma-Aldrich, United Kingdom).

Molecular Biology

The CXCR4 cDNA sequences were provided through the ONCORNET consortium from Vrije Universiteit Amsterdam in pcDEF3 plasmids. pCDNA3.1 (+) neo expression constructs encoding SNAP/CXCR4 were generated as described previously (White et al., 2020), except that sig-SNAP (Gherbi et al., 2015) was ligated in frame using the restriction enzymes BamHI and XhoI in place of sig-NLuc.

CRISPR/Cas9 genome engineering

Guide RNA construction was performed as described previously in the detailed protocol (Ran et al., 2013). Briefly, guide sequences were designed using the CRISPR Design Tool (http://crispr.mit.edu/) to target the C-terminus of CXCL12 (ACTTGTTTAAAGCTTTCTCC) and ligated as complementary oligonucleotides 5’-AAACGGAGAAAGCTTTAAACAAGTC-3’ and 5’-CACCGACTTGTTTAAAGCTTTCTCC-3’ into the pSpCas9(BB)-2A-Puro (PX459 V2) expression construct (from Feng Zhang, Addgene plasmid # 62988) linearized by the restriction enzyme BbsI (NEB). To introduce DNA encoding GSSG-HiBiT into the CXCL12 genomic locus, a donor repair template was designed using the human genome assembly (GRCh38/hg38) and UCSC genome browser (http://genome.ucsc.edu/). The repair template was synthesised as single stranded oligo DNA nucleotides (ssODN, Integrated DNA Technologies, Inc. (IDT)) and consisted of homology arms surrounding GSSG-HiBiT with the CXCL12 stop codon deleted and the PAM motif mutated by a silent C-T substitution. The sequence used was 5’-GAACAACAACAGACAAGTGTGCATTGACCCGAAGCTAAAGTGGATTCAGGAGTATCTGGAGAAAGCTTTAAACAAGGGGAGTTCTGGCGTGAGCGGCTGGCGGCTGTTCAAGAAGATTAGCTAAGCACAACAGCCAAAAAGGACTTTCCGCTAGACCCACTCGAGGAAAACTAAAACCTTGTGAGAGATGAAAGGGCAAA-3’. Positive clones were genotyped using Q5® High-Fidelity DNA Polymerase (New England Biolabs, UK) as per the manufacturer’s instructions and the oligonucleotides 5’-CCTTCCTCCTGTGCAGCC-3’ and 5’-CAGGGTCTAAATGCTGGCAA-3’, which anneal outside the ssODN repair template.

Cell culture

HEK293T cells were maintained in Dulbecco’s Modified Eagle’s Medium (Sigma Aldrich) supplemented with 10% fetal calf serum at 37°C/5% CO_2. Transfections were performed using FuGENE HD (Promega,

USA) according to the manufacturer's instructions. Cells were passaged or harvested using PBS (Sigma Aldrich) and trypsin (0.25% w/v in versene; Sigma Aldrich). CRISPR/Cas9 genome-engineering of HEK293T cells was performed as described previously (Ran et al., 2013; White et al., 2017). Briefly, HEK293T cells were seeded in 6 well plates at 300,000-400,000 cells per well and incubated for 24h at 37°C/5% CO_2. Cells were then transfected with px459 sgRNA/Cas9 expression constructs and the ssODN donor repair template. Cells were cultured for 24h then treated with puromycin (0.3 μg/ml, Sigma-Aldrich) for 3 days to select for transfected cells. Following selection, cells were single cell cloned and allowed to expand for 2-3 weeks. Following expansion single clones expressing CXCL12-HiBiT were screened for luminescence following the addition of furimazine (10 μM) and purified LgBiT (10 nM) using a PHERAStar FS plate reader (BMG LabTech).

CXCL12-HiBiT assays

To investigate CXCL12-HiBiT secretion, wildtype or HEK293 cells expressing genome-edited CXCL12-HiBiT were seeded into poly-D-lysine coated white flat bottom 96 well plates at 30,000 or 60,000 cells/well and incubated for 24h at 37°C/5%CO_2. On the day of the assay, cells were washed and incubated with 50 μL/well pre-warmed 1x HEPES Buffered Salt Solution (1xHBSS; 25mM HEPES, 10mM glucose, 146 mM NaCl, 5 mM KCl, 1 mM $MgSO_4$, 2mM sodium pyruvate, 1.3mM $CaCl_2$, 1.8g/L glucose; pH 7.2) supplemented with 0.1% BSA for 1, 2 or 4 hours or immediately incubated with purified LgBiT (30 nM) and furimazine (10 μM). Total luminescence was then measured on a PHERAStar FS plate reader. Analysis of the effect of time and cell number on CXCL12-HiBiT expression was performed at 20 minutes post LgBiT addition. In assays to investigate the effect of glycosaminoglycan or CXCR4 modulation on the levels of observable CXCL12-HiBiT, cells were washed with HBSS then incubated with HBSS supplemented with 0.1% BSA and 30 nM purified LgBiT for 2h 37°C. Furimazine (10 μM) was added to cells and allowed to equilibrate for 5 minutes before total luminescence was measured on a PHERAStar FS plate reader and 5 basal reads were taken. At time = 0, HBSS, AMD3100 (1 μM), surfen (10 μM), heparan sulfate (30 μg/mL), AMD3100 (1 μM) plus heparan sulfate (30 μg/mL) or surfen (10 μM) plus heparan sulfate (30 ug/mL) were added to the wells and total luminescence was measured. Baseline corrected luminescence was calculated by subtracting vehicle (HBSS)-treated luminescence from the ligand-treated luminescence.

Quantification of tagged protein by luciferase activity

To quantify CXCL12-HiBiT expression, wildtype HEK293 cells or HEK293 cells expressing genome-edited CXCL12-HiBiT were seeded into poly-D-lysine coated white flat bottom 96 well plates at 30,000 or 60,000 cells/well and incubated for 24h at 37°C/5% CO_2. On the day of the assay, genome-edited CXCL12-HiBiT HEK293 cells were washed and incubated with pre-warmed HBSS for 2h at 37°C. A log HiBiT control protein (HiBiT-HaloTag, Promega, USA) standard curve (1 fM - 1 nM) was constructed in parallel by diluting the purified HiBiT control protein in HBSS supplemented with 0.1% BSA and adding to wells containing wildtype HEK293 cells. Purified LgBiT (100 nM) was then added to each well and cells incubated for a further 5 minutes before 10 μM furimazine was added and total light emissions were measured on a PHERAStar FS plate reader. Total assay volume was 50 μL/well.

CXCL12-HiBiT NanoBRET ligand binding

For CXCL12-HiBiT NanoBRET competition ligand binding assays, wildtype HEK293 cells were seeded in 6 well plates at 300,000 cells per well and incubated for 24h at 37°C/5% CO_2. Cells were then transfected with 500 ng/well pcDNA3.1 (neo) plasmid encoding SNAP/CXCR4 and incubated for a further 24h. Transfected or un-transfected HEK293 cells were then seeded with HEK293 cells expressing genome-edited CXCL12-HiBiT into poly-D-lysine coated white flat bottom 96 well plates, at 20,000 cells/well of each cell line and incubated for 24h at 37°C/5% CO_2. On the day of the assay, cells were incubated with 0.25 μM membrane impermeant SNAP-surface AF488 for 1h at 37°C/5% CO_2 prepared in 100 μL/well serum-free DMEM. After incubation, cells were washed 3 times with pre-warmed HBSS and incubated with purified 30 nM purified LgBiT in the absence or presence of AMD3100 (1 pM – 10 μM) for 2h at 37°C with a total assay volume of 50 μL/well. Following ligand incubation, 10 μM furimazine was added and plates equilibrated for 5 mins at room temperature. Sequential filtered light emissions were recorded using a PHERAStar FS plate reader using 475 nm (30 nm bandpass) and 535 nm (30 nm bandpass) filters. BRET ratios were calculated by dividing the 535 nm emission (acceptor) by the 475 nm emission (donor).

Determination of CXCL12-HiBiT-LgBiT affinity

To investigate the affinity of CXCL12-HiBiT-LgBiT complementation, HEK293 cells expressing genome-edited CXCL12-HiBiT or wildtype HEK293 cells were seeded into poly-D-lysine coated white flat bottom 96 well plates at 30,000 cells/well and incubated for 24h at 37°C/5% CO_2. On the day of the assay, cells were washed and incubated with HBSS supplemented with 0.1% BSA for 2hrs at 37°C. Cells expressing genome-edited CXCL12-HiBiT were then incubated with increasing concentrations of purified LgBiT for 30 minutes at 37°C in the absence or presence of surfen (10 μM). In parallel, non-specific luminescence was determined by adding purified LgBiT to wells containing wildtype cells only. Total assay volume was 50 μL/well. Following incubation, furimazine (10 μM) was added, plates incubated for 5 minutes, and total light emissions measured using a PHERAStar FS plate reader. In a subset of experiments on the day of assay cells were washed and incubated with 50 uL/well HBSS supplemented with 0.1% BSA for 2hrs at 37°C before 30 uL of supernatant was collected and transferred to a new white flat bottom 96 well plate and a plate back added. Supernatants were then incubated with increasing concentrations of purified LgBiT for 30 minutes at 37°C in the absence or presence of surfen (10 μM), or Heparan sulfate (30 μg/ml). Before total light emissions were measured as described above. Total assay volume was 50 μL/well and non-specific luminescence was determined by adding purified LgBiT to wells containing supernatant from wildtype cells only. The effect of surfen or heparan sulfate on the affinity complementation of purified HiBiT and LgBiT was determined as described previously using a 50 μL/well total assay volume (White et al., 2020).

Data presentation and statistical analysis

BRET ratios were calculated by dividing the acceptor emission by the donor emission. Calculation of baseline corrected BRET ratios or luminescence values are described in the methods for each assay configuration.

Prism 7 software was used to analyse ligand-binding curves. For CXCL12-HiBiT – purified LgBiT saturation complementation assays, total and non-specific saturation binding curves were simultaneously fitted using the following equation:

$$BRET\ Ratio = \frac{Bmax * [B]}{[B] + KD} + ((M * [B]) + C)$$

where Bmax is the maximal response, [B] is the concentration of LgBiT in nM, KD is the equilibrium dissociation constant in nM, M is the slope of the non-specific binding component and C is the intercept with the Y-axis. Luminescence generated by LgBiT [B] incubated on wildtype cells was used as non-specific binding.

Inhibition concentration response-data were fitted using the following equation:

$$Inhibition = \frac{Bmax - B}{(\frac{[L]}{IC50}) + 1} + B$$

where Bmax is the maximum response of the probe, B is the non-specific binding or response, with both Bmax and B defined from the plateaus of the curve. [L] is the concentration of the competing ligand, IC50 is the concentration of the competition ligand required to inhibit 50% of the maximum response. pIC_{50} values were calculated as -log IC_{50}.

Quantification of HiBiT-CXCL12 expression was interpolated by Prism from linear regression of a log-log standard curve fitted with the following equation:

$$Y = A + B[X]$$

where [X] is the concentration of HiBiT, Y is the luminescence output, A is the y-intercept and B is the slope of the line. Complementation of HiBiT-LgBiT has been reported to produce a linear correlation extending over 8 orders of magnitude (Schwinn et al., 2018). Here we observed a linear correlation of $R^2 = 0.99$, with a slope of 0.85 ± 0.04 (n=6). Statistical analysis was performed using Prism 7 software (GraphPad, San Diego, USA) using one or two-way ANOVA with appropriate multiple comparisons tests where required. Specific statistical tests used are indicated in the figure legends and were performed on the mean data of individual experiments (n) also indicated in the figure legends. A p-value <0.05 was considered statistically significant.

Supplemental References

Gherbi, K., May, L.T., Baker, J.G., Briddon, S.J., Hill, S.J., 2015. Negative cooperativity across beta1-adrenoceptor homodimers provides insights into the nature of the secondary low-affinity CGP 12177 beta1-adrenoceptor binding conformation. FASEB J 29, 2859–2871.

Ran, F.A., Hsu, P.D., Wright, J., Agarwala, V., Scott, D.A., Zhang, F., 2013. Genome engineering using the CRISPR-Cas9 system. Nat Protoc 8, 2281–2308.

Schwinn, M.K., Machleidt, T., Zimmerman, K., Eggers, C.T., Dixon, A.S., Hurst, R., Hall, M.P., Encell, L.P., Binkowski, B.F., Wood, K. V, 2018. CRISPR-Mediated Tagging of Endogenous Proteins with a Luminescent Peptide. ACS Chem Biol 13, 467–474.

White, C.W., Caspar, B., Vanyai, H.K., Pfleger, K.D.G., Hill, S.J., 2020. CRISPR-Mediated Protein Tagging with Nanoluciferase to Investigate Native Chemokine Receptor Function and Conformational Changes. Cell Chem. Biol. 27, 499-510.

White, C.W., Vanyai, H.K., See, H.B., Johnstone, E.K.M., Pfleger, K.D.G., 2017. Using nanoBRET and CRISPR/Cas9 to monitor proximity to a genome-edited protein in real-time. Sci. Rep. 7, 3187.
